# Enamel Analysis by 3D Scanning after Three Orthodontic Clean-Up Procedures: An In-Vitro Test of a New Piezoelectric Tool

**DOI:** 10.3390/ijerph20032516

**Published:** 2023-01-31

**Authors:** Gianna Maria Nardi, Marta Mazur, Roberta Grassi, Serena Rifuggiato, Vito Stiuso, Joanna Janiszewska-Olszowska, Livia Ottolenghi, Ersilia Barbato, Paolo Minetola, Luca Iuliano

**Affiliations:** 1 Department of Odontostomatological and Maxillofacial Sciences, Sapienza University of Rome, 00161 Rome, Italy; 2 Department of Oral Surgery, Tor Vergata University, 00100 Rome, Italy; 3 Department of Management and Production Engineering, Politecnico di Torino, 10129 Torino, Italy; 4 Department of Interdisciplinary Dentistry, Pomeranian Medical University in Szczecin, 70-111 Szczecin, Poland

**Keywords:** prevention, orthodontic clean-up, orthodontic debonding, 3D scanner, residual adhesive removal, enamel damage

## Abstract

(1) Background: To assess the clinical safety and efficacy of a new piezoelectric instrument for orthodontic clean-up; (2) Methods: An in-vitro comparative study on 75 teeth extracted for orthodontic reasons compared the tested method (Treatment 1) with two other procedures: One step finisher and polisher (Inverted cone One gloss Shofu Dental, Kyoto, Japan) (Treatment 2) and twelve-fluted tungsten carbide bur (123-603-00, Dentaurum, Pforzheim, Germany) and Sof-Lex discs Pop-On XT Kit (3M ESPE) (Treatment 3), with n:25 samples in each group. Clinical safety (enamel volume loss) and effectiveness (residual adhesive volume) were assessed using the structured light 3D scanner Atos Compact Scan (GOM GmbH) together with the support of Atos Professional software. The surfaces were scanned three times to assess: (i) the volume of the residual adhesive (RAV) after bracket removal; (ii) the volume of the relative residual adhesive (*dAV*) after the clean-up procedure; (iii) volume of the enamel loss (EVL); (3) Results: The mean RAV (mm^3^) was 0.239 ± 0.337; 0.069 ± 0.124, 0.120 ± 0.193 and the mean EVL (mm^3^) was 0.1870 ± 0.177, 0.187 ± 0.299 and 0.290 ± 0.205, for treatment 1, 2 and 3, respectively. The distribution was asymmetrical between groups in both cases; (4) Conclusions: The tested instrument proved to be effective and safe for post-orthodontic clean-up. With the increasing use of invisible aligners, the possibility of using an ergonomic and fast instrument is of benefit to both patient and practitioner.

## 1. Introduction

Removing a fixed orthodontic appliance consists of the following procedures: debonding brackets, removing the remaining adhesive (orthodontic clean-up) and polishing [1]. Debonding may be performed using bracket removal pliers, side-cutting pliers, lift-off debracketing pliers or air-pressure pulse devices [2].

The bonding resin infiltrates etched enamel into various depths depending on the use of 37% phosphoric acid or a self-etching system [3,4,5]. Therefore, bond failure occurring on the interface between composite and enamel results in enamel damage detectable in direct scanning with blue light technology [6]. Thus, it seems safer to the enamel if the bond failure occurs between the bracket and bonding material. Then, the remaining adhesive must be mechanically removed. Unfortunately, no technique allows performing orthodontic clean-up without enamel damage [1].

In the event of improper procedure, removing a fixed orthodontic appliance may result in:

(i) leaving adhesive remnants on the tooth surface that may become discoloured over time as a result of environmental exposure to food and lifestyle dyes [7]. The adhesive remnants left on the surfaces of clinical crowns predisposes to the accumulation of the oral cavity biofilm. In both cases, this results in aesthetic discomfort for the patient, as well as an increased risk of carioreceptivity [8].
(ii) iatrogenic enamel damage resulting from an excessively aggressive procedure which, in addition to removing the composite, causes quantitative damage to the enamel surface, such as enamel cracking and fracture, removal of the external layer of enamel rich in fluoride and roughening of the surface [6]. Especially in the anterior sectors, this can cause aesthetic damage as the quaternary anatomy of the tooth morphology is cancelled, as is the perikymata, which defines aesthetic enamel details such as light refraction in the juvenile dentition [9,10,11].

A systematic review by Janiszewska-Olszowska et al. [1], aiming to provide evidence for orthodontic adhesive removal methods, showed that the most used rotary instruments were tungsten carbide burs, due to their clinical effectiveness and speed, compared to others available on the market, such as rubbers, Sof-Lex discs or composite burs, ultrasonic tools hand instruments. However, it was emphasised that carbide burs also removed an important layer of enamel and scratched the surface, but to a lesser extent compared to Arkansas stones, green stones, diamond burs, steel burs, and lasers [1].

It has been found that using ultrasound before debonding reduces the force required to debond a bracket [12]. In the study by Santana et al., 2016, applying an ultrasound tip for two seconds at each bracket side on the adhesive bond interface before bracket debonding reduced shear bond strength [12]. Moreover, the use of an ultrasound probe for the removal of remaining glassionomeric cement used for bracket bonding was described by the authors of [13,14,15], who used an ultrasonic instrument to remove the residual adhesive after debonding; however, no objective quantitative instrumental method was used to assess the enamel surface.

The methodology traditionally used in the last four decades to investigate the effectiveness and safety of orthodontic debonding methods, was to take impressions and models of the treated enamel surfaces, and then analyse them using electron microscopy systems [16,17]. However, the need to duplicate the enamel surface, using very precise materials such as impression silicone and very hard plaster for models, resulted in reduced accuracy of the measurements themselves. This step was not overcome even with white light 3D scanners due to the refraction caused by the enamel surface [18]. 

The methodological innovation introduced by the use of blue light 3D scanners finally avoided the need to replicate the treated enamel surface and significantly increased the accuracy of the measurements, allowing comparative clinical studies between different methods used for orthodontic clean-up [6,19].

Given that the orthodontic clean-up procedure is a very important and investigated phase of orthodontic therapy itself, the aim of this comparative in vitro study using a blue light laser scanner was to: (i) study two new piezoelectric inserts to evaluate a novel minimally invasive clinical debonding approach; (ii) evaluate the safety of these procedures for the enamel surface in terms of the volume of enamel removed.

## 2. Materials and Methods

### 2.1. Study Design and Setting

The study was conducted in collaboration between Sapienza University in Rome, the Department of Oral and Maxillofacial Sciences and the Politecnico di Torino, Department of Management and Production Engineering.

### 2.2. Specimen Preparation (Inclusion Criteria)

The test specimen consisted of 75 third molars extracted for orthodontic reasons. The buccal surfaces of the teeth had to be intact and free from enamel cracks, carious processes ICDAS II score 1, 2, 3, 4, 5, 6 and without the presence of composite or other materials restorations. The teeth were kept hydrated at 4 °C for no longer than two weeks. They were stored in distilled water for 24 h prior to orthodontic bonding. The surfaces were then cleaned with a prophylactic brush and a non-abrasive, fluoride-free paste, rinsed for 10 s, and then dried with an oil-free compressed air stream. The teeth were placed one by one in niches on a rack embedded in impression silicone (V-Posil starter set Putty Fast, VOCO GmbH, Cuxhaven, Germany) to avoid movements and even minimal changes in position.

### 2.3. Sample Size Calculation

Sample sizes were determined for the probability of the Type I error α = 0.05, the power 1 − β = 0.8. Based on data in [20] typical mean difference was taken equal to 0.006, standard deviation—to 0.007. This gives the standardized effect size (ratio of mean difference to standard deviation) equal to 0.86, and n = 22 in each group [21]. 

### 2.4. Bonding and Debonding Procedures

The surface treatment was carried out as follows: etching with 35% phosphoric acid gel (Vococid, VOCO GmbH, Cuxhaven, Germany) for 20 s, removal of the acid and cleaning of the residue with an air–water spray, application of the chemical-cure orthodontic adhesive (BrackFix Primer, VOCO GmbH, Cuxhaven, Germany), placement of the molar tubes (Direct Bond Tubes, American Orthodontics, Sheboygan, WI, USA) on the base of which the resin (BrackFix Adhesive, VOCO GmbH, Cuxhaven, Germany) was previously placed. The molar tube was placed on the buccal surface with slight pressure in order to avoid thickening of the resin, excess around the molar tube was removed using a micro-brush.

Then, after waiting about 10 min, the teeth were kept for 24 h in a 0.9% saline solution and then rinsed with distilled water to prevent crystallization. To remove the molar tubes from the buccal dental surface, ligature cutting pliers were used, positioned along the mesiodistal surface of the clinical crown, in order to simulate what is performed in clinical reality on the patient.

### 2.5. Clean-Up Procedures

The total sample of 75 teeth was distributed in three study groups according to the procedure used to remove the adhesive remnants:

1. Group 1: Tested treatment inserts: ultrasonic scaler (Multipiezo device; Mectron, Italy), with the following inserts:
  - Insert diamond grain 30 µm, made of medical grade stainless steel
  - Insert made of medical grade stainless steel with an operative part made of modified PEEK.
2. Group 2: One step finisher and polisher (Inverted cone One gloss Shofu Dental, Kyoto, Japan)
3. Group 3: Twelve-fluted tungsten carbide bur (123-603-00, Dentaurum, Pforzheim, Germany) and Sof-Lex discs Pop-On XT Kit (3M ESPE). The tungsten carbide bur on a low-speed handpiece at 20,000 rpm without water cooling, and a new bur was used after 12 teeth. The Sof-Lex discs system (medium) was used under dry settings with light pressure for 20 s at a handpiece speed of 10,000 rpm.

All three procedures were performed by the same operator, an experienced orthodontic dentist using a 4.5X magnification system. Time was standardized to 30 s/tooth for each treatment or each treatment step.

### 2.6. 3D Optical Analysis

The scanning of the rack with the specimens was performed through a non-contact blue-light 3D scanner in three steps which were named T0, T1 and T2 according to the condition of the teeth:

(i) T0 represents the first phase and refers to the original buccal surface of clinical crowns at baseline.
(ii) T1 is the buccal surface condition after mechanical removal of the molar tubes with orthodontic pliers; at this time of follow-up the adhesive remnants were measured.
(iii) T2 represents the last step, and it refers to the cleaned buccal surface, after the three tested procedures for removing adhesive remnants.

**Figure 1:**
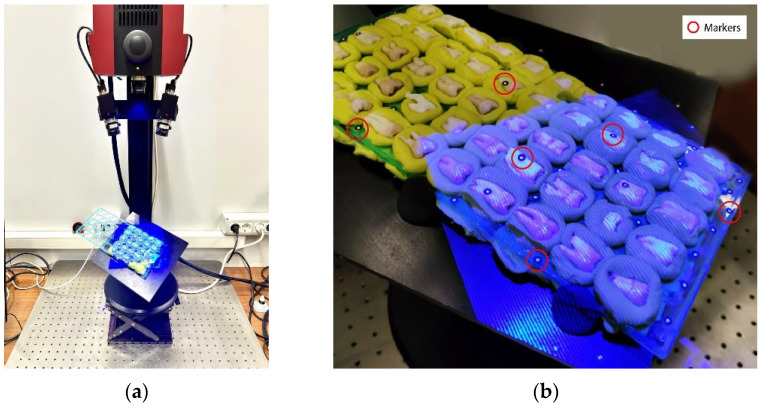
(**a**) GOM Atos Compact Scan. (**b**) Scanning process by structured light fringes with indication of some markers.

The scanning process was carried out using the structured light 3D scanner Atos Compact Scan (Figure 1a) by the German company GOM GmbH together with the support of Atos Professional software version 2020 (GOM, Braunschweig, Germany). The scanner has a central projector to project blue-light fringes on the scanned object and two lateral cameras for the stereoscopic vision. Atos Compact Scan has a measuring volume of 125 mm × 90 mm × 90 mm with a resolution of 2 megapixels for the cameras. Therefore, it can acquire 2 million points spaced about 0.075 mm per single scan. Its metrological performance is certified by VDI/VDE 2637 German standard and the maximum measuring error is below 20 µm.

The preliminary phase of the scanning procedure consisted in preparing accurately the rack by fixing it on a rotating platform using some plasticine and by positioning on it some adhesive stickers named markers, as shown in Figure 1b. The aim of the markers, which have a white circle on a black background, is to create a net of fixed reference points on the scanned object. The reference points, which are represented by the centre of the white circles, are used by Atos Professional software (GOM, Braunschweig, Germany) to automatically align multiple scans of the rack, which are acquired from different viewpoints. Since the rack’s overall dimensions exceed the working area of the scanner, it was necessary to rotate and tilt the platform to scan all the buccal surfaces in a sequence of multiple scans. After the acquisition phase of T0 condition, the rack was removed from the platform so that molar tubes could be applied and debonded to reach T1 condition, as explained in the previous paragraph.

Because of the presence of the markers, the scanning software Atos Professional (GOM, Braunschweig, Germany) could recognize the fixed reference points and align the new scan data and measurement series to the one in the T0 condition. Once the first scans in the T1 condition were acquired, the previous scan data of the T0 condition was deleted to complete the acquisition of the rack in the T1 condition. Then, the T2 condition was obtained by removing the adhesive remnants through the three tested procedures. Finally, the same scanning procedure was applied to align and refer scan data of the T2 condition to the original T0 condition.

### 2.7. Data Alignment for Comparison

After completing the scanning phase, the scan data was processed to compare the three clean-up procedures for each different tooth. First of all, the scan data corresponding to the surface of each single tooth was extracted from the data of the entire rack for T0, T1 and T2 conditions. Then, the data in the T1 and T2 conditions were aligned to that in the T0 condition by applying a best-fit operation, which corrects smaller systematic errors by computing a roto-translation matrix that minimizes data deviations. The best fitting, which was carried out by GOM Inspect software version 2020 (GOM, Braunschweig, Germany), requires the manual selection of the corresponding areas of each tooth that were not affected by the bonding and debonding operations, i.e., the crown and roots. After best fitting, the aligned scan data of each tooth was classified and exported in STL format for T0, T1 and T2 condition.

**Figure 2:**
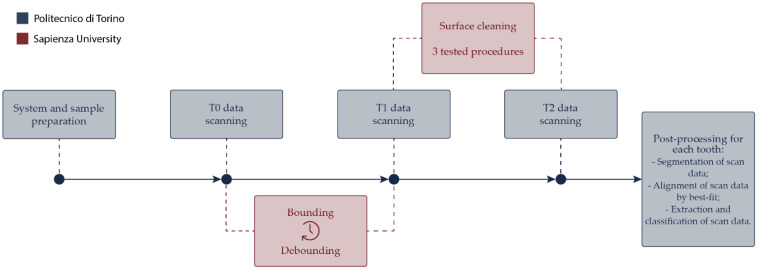
Steps for scan data acquisition.

A summary of the described scanning procedures is illustrated in Figure 2.

Assuming the scan data of the T0 condition as the reference data, the scan data of the T1 condition was compared to it to compute the adhesive volume after the bonding and debonding operations. The scan data of the T2 condition was compared to the T0 condition to evaluate the residual adhesive volume and the volume of enamel loss after the clean-up procedure.

### 2.8. Data Comparison

The comparisons and data analysis were carried out using Rapidform 2006 software (INUS Technology, Seoul, South Korea) that is certified by the German Metrological Institute Physikalisch-Technische Bundesanstalt (PTB) relative to computational algorithms and validity of numerical results provided by the generation of geometries and measurements from scan data.

**Figure 3:**
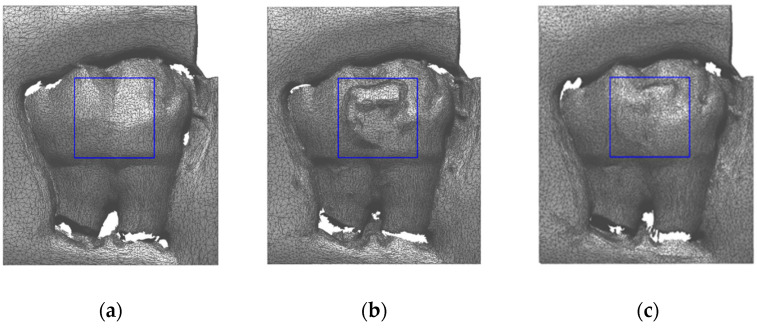
Cube positioning (blue frame) on tooth 21 in T0 condition (**a**), T1 condition (**b**), and T2 condition (**c**).

The data comparisons were limited to an analysis area of 5 mm × 5 mm on the top view of each tooth. The extension of the area was chosen to use a comparable data set with a similar number of scan points on the surface of all 75 teeth. This area was positioned ad-hoc for each tooth in the T1 condition to cover entirely the zone of bracket application (Figure 3). Hence, the area also includes the residual adhesive volume and the enamel loss in the T2 condition.

**Figure 4:**
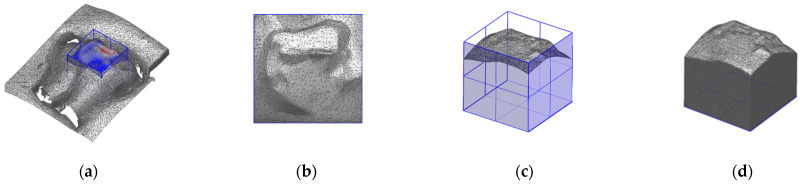
Cube positioning (blue frame) in T1 condition on tooth 21 (**a**); cut surface of T1 condition in top view (**b**); cut surface in iso view with cube planes (**c**); closed shell (**d**).

To trim the area of analysis, a cube with a side length of 5 mm was positioned in T1 data. The cube positioning was carried out manually by the human operator with the aid of the deviation map between the T1 surface data and the T0 reference data (Figure 4a). The cube’s lateral faces were used as cutting planes in order to isolate the defined area and delete the external scan data. Figure 4b shows the resulting cut area of interest from the top view of the tooth. Starting from this result (Figure 4c), closed shells were created for all conditions T0, T1 and T2 of the single tooth. Each shell was generated by extrusion of the side edges of the area of analysis to reach the bottom plane of the cutting cube (Figure 4d). 

Therefore, the closed shells for T0, T1 and T2 conditions have the same bottom and side surfaces, whereas the top surface, which corresponds to the area of analysis, is different. This preliminary step was repeated for each individual tooth generating a total of 225 closed shells. 

Magics software version 22.03 by Materialise (Leuven, Belgium) was used to evaluate the differences between the closed shells in STL format of each tooth. Through simple Boolean operations, it was possible to quantitatively assess the amount of adhesive in the T1 condition, the amount of residual adhesive and the amount of enamel loss in the T2 condition. The T0 condition was assumed as the reference closed shell and the following Boolean operations were carried out:

(i) Subtraction of T0 closed shell from T1 closed shell to extract the volume of adhesive after bracket removal.
(ii) Subtraction of T0 closed shell from T2 closed shell to extract the volume of the residual adhesive after the clean-up procedure.
(iii) Subtraction of T2 closed shell from T0 closed shell to extract the volume of the enamel loss.

**Figure 5:**
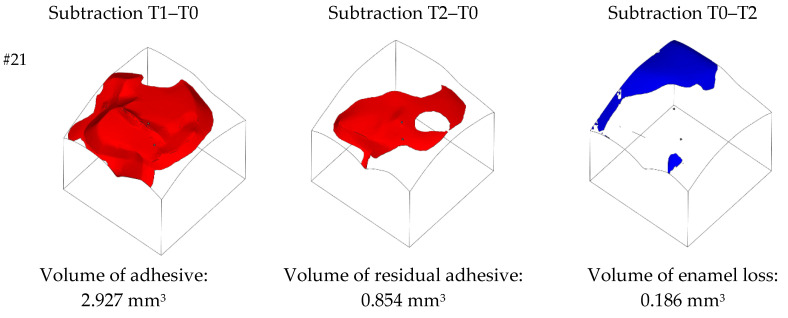

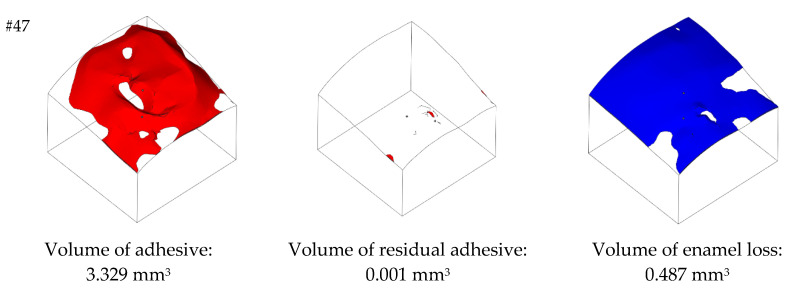
Examples of Boolean operations and volume extraction. Red volumes represent the residual adhesive, while blue volumes represent the enamel loss with respect to T0 condition.

Boolean subtraction operations were performed by setting a clearance of 0.020 mm to account for the maximum measuring error of Atos Compact Scan according to VDI/VDE 2637 German standard. Examples of the Boolean operations and resulting volume values are reported in Figure 5 for the inspected teeth 21 and 47.

### 2.9. Statistical Analysis

ANOVA with Tukey HSD post hoc test was used to check the difference between three treatments. The normality of distributions was checked using the Shapiro–Wilk test. The results were considered statistically significant at *p* < 0.05. The R statistical program, ver. 4.1.2 (The R Foundation for Statistical Computing, Wirtschaftsuniversität Wien, Vienna, Austria) was used for the statistical analyses. 

## 3. Results

### 3.1. Results (Difference in Treatment Efficacy)

#### 3.1.1. Residual Adhesive Volume

Descriptive statistics for residual adhesive volume in the T1 condition are reported in Table 1.

**Figure 6:**
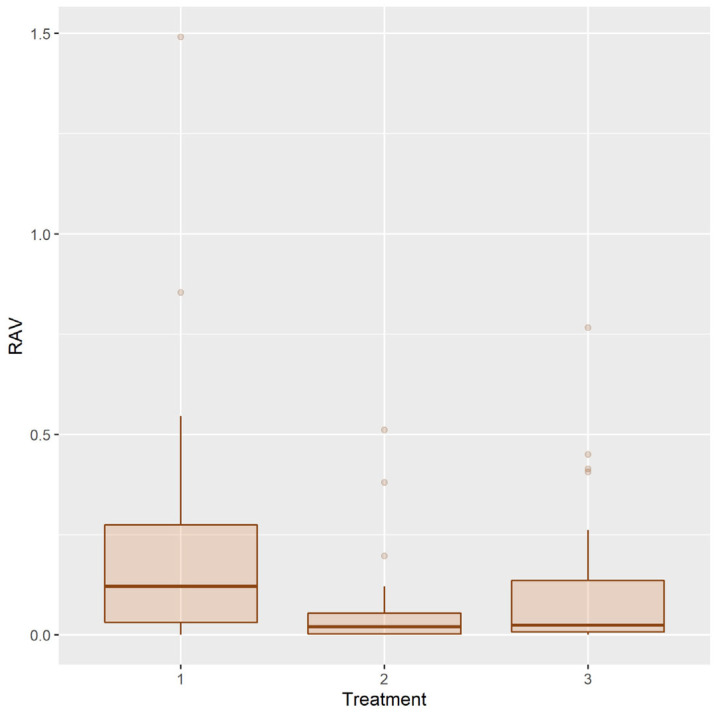
Distribution of the residual adhesive volume after treatments 1, 2, 3.

The efficacy of treatments was compared both using absolute amount of residual volume and relative to adhesive volume at T1. The distribution of the residual adhesive volume (RAV) after treatments is shown in Figure 6.

**Figure 7:**
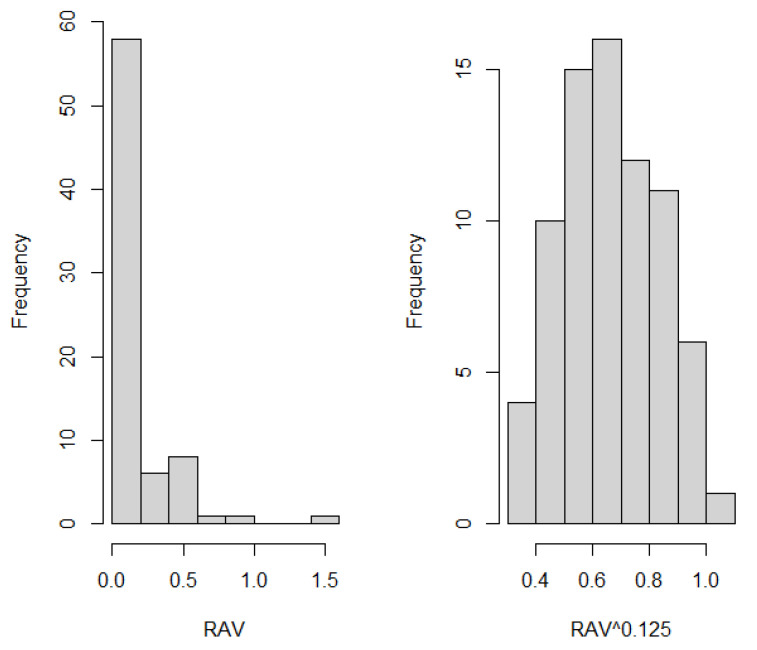
Histograms of RAV (**left**) and RAV^0.125^ (**right**).

RAV has very asymmetrical distribution in all three treatment groups. To achieve normality of distribution, power function transformation was used. With power 0.125, data does not reveal statistically significant difference from normal distribution (*p* = 0.375, 0.341, 0.353 in groups 1, 2, 3, respectively). Histograms of original and transformed RAV are shown in Figure 7.

ANOVA reveals significant difference in efficacy of treatments (F [2, 72] =3.565, *p* = 0.033).

The Tukey HSD post hoc test stated that a statistically significant difference occurs between treatments 1 and 2 only (Table 2). The efficacy of treatment 1 is lower than that of treatment 2. 

Relative residual adhesive volume was examined beside absolute amount of RAV. Variable

(1) $$dAV=\frac{\mathrm{Residual}\;\mathrm{adhesive}\;\mathrm{volume}}{\mathrm{Adhesive}\;\mathrm{volume}\;\mathrm{at}\;T1}$$

was introduced. The variable indicates what part of the initial (at T1) adhesive volume remains after treatment. 

**Figure 8:**
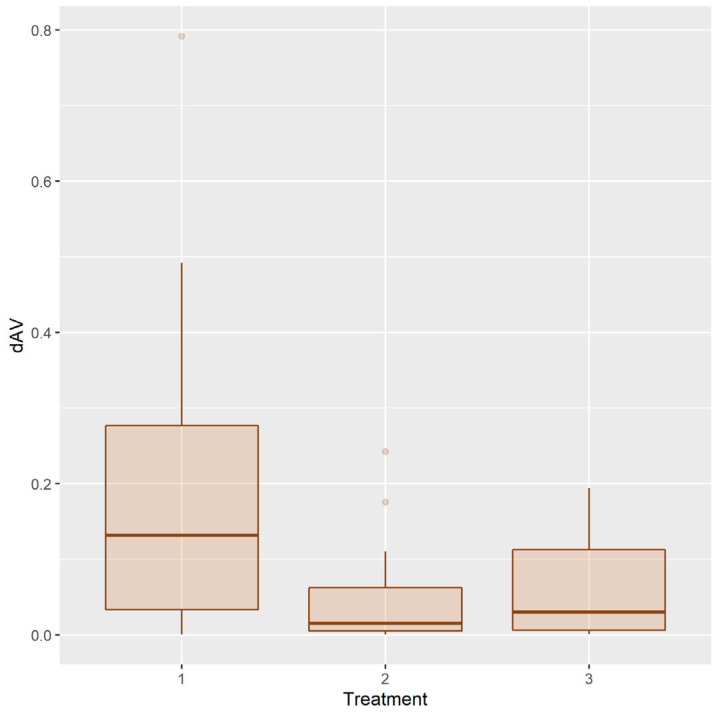
Distribution of the relative residual adhesive volume after treatments 1, 2, 3.

The distribution of the relative residual adhesive volume (*dAV*) in the T2 condition after different treatments is shown in Figure 8.

Power function transformation was used also to achieve normality of distribution. With power 0.225, the data do not reveal a statistically significant difference from normal distribution (*p* = 0.273, 0.306, 0.053 in groups 1, 2, 3, respectively).

ANOVA reveals a significant difference in efficacy of treatments (F [2, 72] =6.921, *p* = 0.002).

The Tukey HSD post hoc test stated that a statistically significant difference occurs between treatments 1 and 2, and treatments 1 and 3 (Table 3). The efficacy of treatment 1 is lower compared both to treatment 2 and treatment 3. 

#### 3.1.2. Enamel Volume Loss

Descriptive statistics for enamel volume loss in the T2 condition are in Table 4.

**Figure 9:**
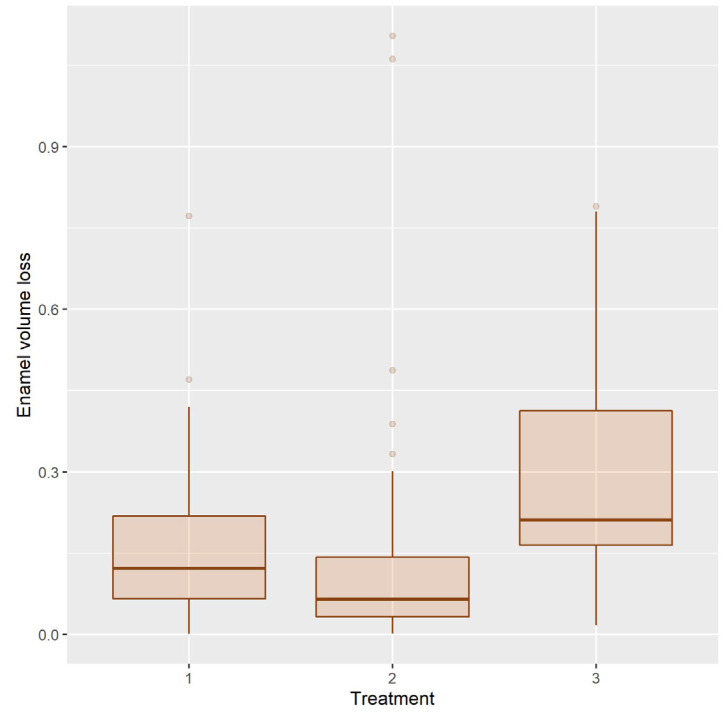
Distribution of the enamel volume loss after treatments 1, 2, 3.

The distribution of the enamel volume loss (EVL) after treatments is shown in Figure 9.

**Figure 10:**
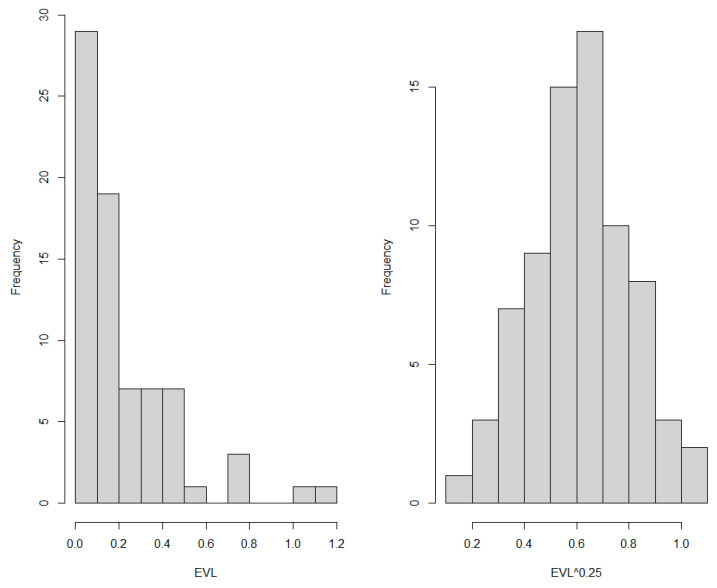
Histograms of EVL (**left**) and EVL^0.25^ (**right**).

EVL has very asymmetrical distribution in all three treatment groups. To achieve normality of distribution, power function transformation was used. With power 0.25, the data do not reveal a statistically significant difference from normal distribution (*p* = 0.980, 0.086, 0.231 in groups 1, 2, 3, respectively). Histograms of original and transformed EVL are shown in Figure 10.

ANOVA reveals a significant difference in efficacy of treatments (F [2, 72] =4.591, *p* = 0.013).

The Tukey HSD post hoc test stated that a statistically significant difference occurs between treatments 2 and 3 (Table 5). The efficacy of treatment 1 does not differ significantly from treatments 2 and 3. 

## 4. Discussion

This in vitro study was designed to analyse the efficacy and clinical safety of three different orthodontic debonding procedures. One of these procedures was experimental, the other two served as comparison.

The samples selected for the study were third molars, removed for orthodontic reasons, free from reconstruction, caries and other damage, such as cracks on the enamel surface.

The current study is one of the few in the literature in which the technology of blue light 3D scanning was used, which allows for a very high level of accuracy, and to calculate, through three consecutive scans, the adhesive volumes in T1, in T2, which allow to characterize the clinical effectiveness of the tested method and the enamel volume between T2 and T0, which allow us to describe the safety of the method each time.

In 2014, in a study by Janiszewska-Olszowska J et al. [6], the enamel surface was evaluated for the very first time with blue light 3D technology before and after debonding, but without clean-up, of fifteen third molars. The mean height of composite remnants ranged from 0.0087 mm to 0.238 mm; mean value: 0.084 mm. and the enamel loss ranged from 0.0076 mm to 0.0416 mm, with the deepest enamel loss of 0.2071 mm. Median volume of enamel loss was 0.104 mm^3^ and maximum volume was 1.484 mm^3^ [6]. 

Then, a study by Suliman S. et al. evaluated with a 3D scanner the enamel loss after ceramic bracket debonding and a clean-up using a finishing carbide bur [22,23]. It is not clear the light of the scanner, because no mention of blue light was made in the text. However, the results showed a depth of enamel loss (mean ± SD) post-debond was 21 ± 8 µm and 33 µm and post-cleanup was 28 ± 14 µm and 18 ± 8 µm; the post-debond remnant thickness was 188 ± 113 µm and 120 ± 37 µm (*p* = 0.2381) and post-cleanup was 16 ± 5 µm and 15 µm for polycrystalline and monocrystalline ceramic brackets, respectively. The scanner (COMET xS, Steinbichler Vision Systems, Neubeuern, Germany with an accuracy of 5 µm and a lateral resolution-distance between measured points of 60 µm) and software used in this study did not allow for volume calculation [22].

Then Janiszewska-Olszowska J et al., in 2015, performed an in-vitro analysis using the same 3D blue light technology on 30 molars to assess the amount of remaining adhesive and the clinical safety of three different instruments: One-Step Finisher and Polisher and Adhesive Residue Remover in comparison to tungsten carbide bur. The authors found no statistically significant differences in the volume or mean height of adhesive remnants between the groups. Mean volume of enamel loss was 2.159 mm^3^ for tungsten carbide bur, 1.366 mm^3^ for Shofu One Gloss and 0.659 mm^3^ for Adhesive Residue Remover (*p* = 0.0078) [20]. The latter study was the first to directly measure adhesive remnants and enamel loss resulting from orthodontic clean-up. Moreover, it was also the first study to use One-Step Finisher and Polisher as well as Adhesive Residue Remover in the debonding clean-up [20]. The results of this study are even more promising with a mean volume of enamel loss of 0.180 ± 0.177, 0.197 ± 0.299 and 0.290 ± 0.205, for test treatment, One-step Finisher and Polisher One gloss and twelve-fluted tungsten carbide bur (and Sof-Lex discs, respectively. It is worth saying that orthodontic clean-up is a procedure whose outcome is not only dependent on the tool used and the possibility of working with magnification but is also operator dependent. In turn, studies that are performed to assess efficacy and safety can also be a source of bias resulting from surface topography (fissures and porosity retain more adhesive) and operator-dependent variability in the various phases of the in vitro clinical trial.

It is worth mentioning that in the present study, the procedure of removing adhesive was precisely defined, e.g., limited to 30 s for tooth. Thus, the difference in residual adhesive reflected the effectiveness of different tools. In the study by Janiszewska-Olszowska et al. [20] adhesive was removed “until no macroscopically visible adhesive remnants could be found”. This explains why the results of the present study referring to differences in residual adhesive remnants are not consistent with the study cited. It can be supposed that a longer time could be needed for the less effective tools in the study by Janiszewska-Olszowska et al. [20].

To our knowledge, there is no other in vitro study that used the blue light 3D scanner technology to assess effectiveness and safety of the orthodontic clean-up; for this reason, this study is innovative both in terms of methodology and the information provided as a result of the measurements.

In the past, the methods commonly used to evaluate the clinical safety and efficiency of clean-up procedures in orthodontics were based on contact profilometry, laser scanners and SEM. The clinical design was different case to case, as the analysed objects were human-extracted teeth, plaster models, or epoxy replicas. In addition, the studies with laser 3D white light scanning used plaster models of extracted teeth to reduce light reflections [1]. According to Fitzpatrick and Way [18], it is demonstrated that the measurement error due to silicone impression inaccuracy ranges from −2.5 μm to +3.5 μm. It can also be supposed that model pouring causes a further increase of the measurement error [1].

The test procedure is performed with two new inserts, a pre-market tool:

(a) Insert diamond grain 30 µm, made of medical grade stainless steel.
(b) Insert made of medical grade stainless steel with an operative part made of modified PEEK.

In general, piezoelectric technologies use piezoelectric ultrasonic technology to generate mechanical micro vibrations. The ultrasonic vibration from a piezo scaler is generated from a series of ceramic discs in the handpiece, attached to the device, where the high-frequency vibration (range of 25–35 kHz) is transmitted to the insert.

Rubber wheels may have different abrasive particles and different binders. One Gloss employs aluminium dioxide and silicone dioxide as an abrasive and the abrasive delivery medium is polyvinylsiloxane [24].

A study by Fan XC et al. compared the surface roughness and enamel morphology of teeth after three tools and showed that SEM appearance Cleanup with One-Gloss polisher provided enamel surfaces closest to the intact enamel [25].

On the other side, the most used rotary instruments were tungsten carbide burs, due to their clinical effectiveness and speed, compared to others available on the market, such as rubbers, Sof-Lex discs or composite burs, and ultrasonic tool hand instruments. However, it was emphasised that these burs also removed an important layer of enamel and scratched the surface, but to a lesser extent compared to Arkansas stones, green stones, diamond burs, steel burs, and lasers [1].

An epidemiological study using a questionnaire and conducted among Italian orthodontists, showed that for the orthodontic clean-up, the vast majority used low speed tungsten carbide burs under irrigation (40.08%), then high-speed carbide burs (14.19%), and diamond burs (14.19%). For polishing, rubber cups (36.70%) and abrasive discs (21.35%) were the most common clinical tools [26]. 

## 5. Conclusions

This in-vitro study was carried out to test the efficacy and safety of a new piezoelectric inserts for orthodontic cleaning using innovative technology with a 3D blue light scanner that allowed a high level of accuracy and skipped the need for duplicates of the surfaces to be tested. 

Comparisons were made between two brand-new piezoelectric inserts and the highest standards described and available on the market. The tested tips were evaluated as comparable with the standard tools, being safe and effective in the cleaning procedure.

The new instrument was found to be minimally invasive on the enamel surface.

Considering the widespread use of orthodontic therapies with fixed braces but also therapies with invisible aligners, the results of this study show that the piezoelectric technology is promising for the improvement of clinical ergonomic debonding performance, which allows the preservation of enamel health with the benefits of a minimally invasive debonding procedure.

## Figures and Tables

**Table 1:** Descriptive statistics for residual adhesive volume.

Treatment	n	Min	Max	Mean	SD	Median	95% Conf. Interval
All	75	<0.001	1.491	0.143	0.243	0.043	(0.087, 0.198)
1	25	<0.001	1.491	0.239	0.337	0.121	(0.100, 0.378)
2	25	<0.001	0.511	0.069	0.124	0.02	(0.018, 0.120)
3	25	<0.001	0.766	0.120	0.193	0.024	(0.040, 0.200)

**Table 2:** Tukey HSD post hoc test results for residual adhesive volume.

Treatments	Difference *	*p*
2-1	−0.123	**0.026**
3-1	−0.073	0.259
3-2	0.050	0.535

* The difference of means is for transformed RAV^0.125^ variable. The results were considered statistically significant at *p* < 0.05 (in bold).

**Table 3:** Tukey HSD post hoc test results for relative residual adhesive volume.

Treatments	Difference *	*p*
2-1	−0.184	**0.001**
3-1	−0.125	**0.041**
3-2	0.059	**0.479**

* The difference of means is for transformed *dAV*^0.225^ variable. The results were considered statistically significant at *p* < 0.05 (in bold).

**Table 4:** Descriptive statistics for enamel volume loss.

Treatment	n	Min	Max	Mean	SD	Median	95% Conf. Interval
All	75	0.001	1.104	0.219	0.235	0.143	(0.165, 0.273)
1	25	0.001	0.772	0.180	0.177	0.122	(0.107, 0.252)
2	25	0.002	1.104	0.187	0.299	0.065	(0.064, 0.311)
3	25	0.017	0.79	0.290	0.205	0.211	(0.206, 0.375)

**Table 5:** Tukey HSD post hoc test results for enamel volume loss.

Treatments	Difference *	*p*
2-1	−0.043	0.665
3-1	0.105	0.098
3-2	0.148	**0.012**

* The difference of means is for transformed EVL^0.25^ variable. The results were considered statistically significant at *p* < 0.05 (in bold).

## Data Availability

Dataset is available upon request from the corresponding author.
